# Effect Mechanism of Land Consolidation on Soil Bacterial Community: A Case Study in Eastern China

**DOI:** 10.3390/ijerph19020845

**Published:** 2022-01-13

**Authors:** Yaoben Lin, Yanmei Ye, Shuchang Liu, Jiahao Wen, Danling Chen

**Affiliations:** 1 School of Law and Politics, Nanjing Tech University, Nanjing 211816, China; lyb@njtech.edu.cn; 2 Land Academy for National Development (LAND), Zhejiang University, Hangzhou 310058, China; yymzjuedu@163.com (Y.Y.); 13395775305@163.com (S.L.); 3 Department of Watershed Sciences, Utah State University, Logan, UT 84321, USA; X1525866@163.com; 4 Department of Land Management, College of Public Administration, Huazhong Agricultural University, Wuhan 430070, China

**Keywords:** farmland consolidation, cultivated land quality, soil basic physical and chemical properties, heavy metals, microorganisms

## Abstract

Farmland consolidation is an effective tool to improve farmland infrastructures, soil quality, and sustain a healthy farmland ecosystem and rural population, generating contributions to food security and regional sustainable development. Previous studies showed that farmland consolidation regulates soil physical and chemical properties. Soil microorganisms also play an important role in soil health and crop performance; however, few studies reported how farmland consolidation influence soil microecology. Here, we used DNA sequencing technology to compare bacterial community structure in farmlands with and without consolidation. DNA sequencing technology is the most advanced technology used to obtain biological information in the world, and it has been widely used in the research of soil micro-ecological environment. In September 2018, we collected soil samples in Jiashan County, Zhejiang Province, China, and used DNA sequence technology to compare the bacterial community structure in farmlands with and without consolidation. Our results found that (1) farmland consolidation had significant impacts on soil microbial characteristics, which were mainly manifested as changes in microbial biomass, microbial diversity and community structure. Farmland consolidation can increase the relative abundance of the three dominant bacteria phyla and the three fungal dominant phyla, but it also negatively affects the relative abundance of the six dominant bacteria phyla and the three fungal dominant phyla. (2) Farmland consolidation had an indirect impact on soil bacterial community structure by adjusting the soil physical and chemical properties. (3) The impact of heavy metals on bacterial community structure varied significantly under different levels of heavy metal pollution in farmland consolidation areas. There were 6, 3, 3, and 5 bacterial genera that had significant correlations with heavy metal content in cultivated land with low pollution, light pollution, medium pollution, and heavy pollution, respectively. The number of heavy metal-tolerant bacteria in the soil generally increased first and then decreased under heavy metal polluted conditions. Our study untangled the relationship between varied farmland consolidation strategies and bacteria through soil physcicochemical properties and metal pollution conditions. Our results can guide farmland consolidation strategies and sustain soil health and ecological balance in agriculture.

## 1. Introduction

Farmland consolidation is an effective means to improve farmland infrastructure, improve farmland quality, and protect farmland ecology [1,2]. It is a complex system engineering that plays an important regulatory role in multiple fields such as land ecology, land economy, and land ownership [3,4,5]. Therefore, farmland consolidation has an important contribution to ensuring global food security and regional sustainable development and rural depopulation on a global scale [6,7]. The methods under farmland consolidation mainly include building ditches, merging plots, land levelling, applying organic fertilizers, and comprehensive improvement. A large number of studies have shown that farmland consolidation regulates soil physical and chemical properties and concentrations of heavy metals [6,8,9]. As the most abundant microorganism in the soil environment, soil bacteria are extremely susceptible to varied soil properties [10]. As a key indicator of soil quality and ecological status, soil bacteria play an important role in soil pollution restoration and promotion of crop growth [11,12]. Therefore, it is important to explore how farmland consolidation affects the relative abundance and community structure of bacteria by adjusting soil physical and chemical properties and the content of heavy metals, which can provide information on sustainable development in farmlands.

The farmland conditions can not only affect crop growth and food security, but also have a significant impact on regional climate, hydrology, and soil [13,14,15,16]. Soil bacteria are the key driver of nutrient cyclings and energy flow in the soil environment, contributing to a healthy soil environment, enhanced regional ecosystem service values and ecosystem functions [17]. Soil bacteria are an important part of the earth’s ecosystem, and they affect global climate through the regulation of CO_2_, CH_4_, N_2_O, and other greenhouse gases [18]. Many studies reported the relationship between soil bacteria and climate change, and it has also been confirmed that the regulation of carbon, nitrogen, phosphorus, and other element cycles by soil microorganisms plays a vital role in the feedback of climate change [19,20]. In addition, soil bacteria can promote or inhibit the plant performance and further affect the plant community structure and functions [21,22]. Nitrogen-fixing bacteria in the soil can promote the uptakes of nitrogen (N), phosphorus (P), potassium (K), and other nutrients by plants in the nitrogen-fixing symbiotic relationship. Since N, P, K, and other elements are key nutrients that influence plant growth and reproduction, nitrogen-fixing bacteria can influence the expansion of the growth range of plant communities [23]. Hence, the types and functions of soil bacteria regulate plant community structure and function, as well as evolutionary processes in the ecosystem.

The structure of soil bacterial community is highly susceptible to different soil conditions. As a human activity, farmland consolidation has a strong interference on the physical and chemical properties of the soil, which will directly or indirectly affect the soil bacteria and change its ecological function [8,10,24]. Therefore, the impact of farmland consolidation on soil bacteria will be an important content in the future research fields of land use, environmental management, and microbial diversity [25,26]. Farmland consolidation is one of the most effective land management measures to improve agricultural production and ecological environment, including merging scattered land, improving agricultural facilities and soil quality, which has been widely used in most countries in the world [1,2,16]. Nowadays, in order to cope with the increasing risks of farmland, researchers are exploring the multifunctional potential of farmland consolidation to solve development problems such as agriculture, nature, landscape, economy, and tourism [27,28]. However, farmland consolidation also affects the physical and chemical properties of farmland soil and bacterial communities, especially in terms of soil bacterial diversity [6,27]. In order to promote the strategic deployment of China’s ecological civilization construction, the farmland consolidation is also developing in a more ecological direction. However, the soil microorganisms that are profoundly affected by farmland consolidation have not received sufficient attention and need to be paid enough attention in future research and practice [29]. Under the current situation, it is possible to improve soil quality and increase the productivity of cultivated land by adopting appropriate farmland consolidation methods [30,31]. On this basis, we call to scientifically assess the impact of farmland consolidation on the structure of soil microbial communities, and extract microbial indicators of farmland quality [32,33]. This will be an important topic in the field of global farmland consolidation and ecological environmental protection in farmlands.

However, there were few studies on how farmland consolidation affects soil bacterial characteristics currently, and the mechanism behind them was not clear, which was a blank in the field of farmland consolidation and soil microecology research. Therefore, this study attempted to use experimental methods to explore the influence mechanism of farmland consolidation on soil bacteria in a county of the east coast of China. Specifically, we explored how farmland consolidation affected the relative abundance of bacteria and the community structure by regulating the soil properties and heavy metal contents, established a new research framework for this type of research, and provided information on farmland consolidation measures and ecological restoration in soils.

## 2. Framework and Data Collection

### 2.1. Research Framework

As an engineering method that directly affects arable land, farmland consolidation regulates arable land morphology, land landscape, soil properties, and fertility through measures such as building ditches, merging plots, land levelling, and applying organic fertilizers, combined or respectively [34]. Specifically, the influences of farmland consolidation on soil characteristics include: (1) Increasing the intensity of tillage activities to reduce soil bulk density, increase soil porosity, and improve organic fertilizer uniformity; (2) Applying organic fertilizer to increase soil nutrients availability, optimize the structure of soil microbial community, and improve the structure of soil aggregates and soil fertility; (3) improving farmland irrigation and drainage facilities to ensure the water requirement for crop growth and implement soil moisture management; (4) strengthening the management and protection, standardizing the technical process, and implementing supervision, of farmland consolidation, to sustain soil and ecosystem health [35].

Soil microorganisms are mainly affected by the dual effects of natural environment and human disturbance, including natural factors such as soil nutrients, pH, moisture, soil pollutants, climate change, biological invasion, plant species, as well as farming methods, irrigation management, land use changes and farmland consolidation methods and other human activities [36,37]. This study area is a small-scale farmland soil microbial study with a single farming method, and it has the characteristics of close to natural and local conditions. Therefore, this study limits the influencing factors of soil bacteria to soil properties and farmland consolidation methods to help reveal the true mechanism of action and find effective management strategies.

**Figure 1:**
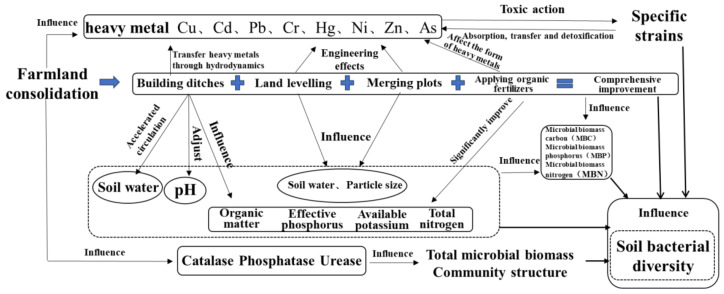
Conceptual diagram illustrating how farmland consolidation influence soil bacteria.

Farmland consolidation is an engineering method that directly affects the soil, including multiple methods such as building ditches, merging plots, land levelling, and applying organic fertilizers. This has a direct effect on the physical and chemical properties of the soil, and it will also directly or indirectly affect the quantity and community structure of soil microorganisms (Figure 1). In addition, through the implementation of mechanical engineering, farmland consolidation can reclaim non-agricultural land into cultivated land, and the infrastructure conditions of agricultural land can also be improved through consolidation projects. The former can lead to heavy metals residue from mines, factories and other lands, and enter the soil to cause pollution. The latter can adjust heavy metal concentrations in the soil through engineering measures, both of which will affect soil bacterial community structure.

In the field of farmland consolidation, few studies have focused on the impact of heavy metal content and basic physical and chemical properties of soil on soil bacteria, and the situation is more complicated in different levels of heavy metal pollution, so further exploration and discussion are needed. Based on the existing research results, this paper puts forward the analytical ideas and empirical research on the changes in soil heavy metal content and the basic physical and chemical properties of soil and the indirect effects of soil microbial characteristics by farmland consolidation, as shown in Figure 1.

### 2.2. Study Area

The study area is located in a county of the east coast of China. The total area of the county is about 500 km^2^, of which the water area accounts for about 15% and the land area accounts for about 85% (Data come from Jiashan County Natural Resources Bureau). The area belongs to the Southeast Asian monsoon region, with four distinct seasons and a mild climate. The annual average temperature is 15.6 °C, the average annual rainfall is 1155.7 mm, the annual average relative humidity is 65%, the annual average insolation duration is about 2000 h, and the frost-free period is 236 days (Data come from Jiashan County Natural Resources Bureau). The county has an advantageous geographical location and pleasant climate, making it an important grain producing area in China. However, the sustainable development of agriculture in this region is being challenged by fragmentation of cultivated land, incomplete agricultural facilities, degradation of soil fertility, and soil pollution. Therefore, farmland consolidation is an important strategy in recent years to improve the agricultural production and soil health in this area.

Through data collection, field investigations, and household interviews, we found that the county’s farmland consolidation methods mainly included four types: building ditches, merging plots, land levelling, and applying organic fertilizers. In the farmland where these four measures were applied, we defined as comprehensive improvement. Collectively, this study divided the county’s farmland into six types: building ditches, merging plots, land levelling, applying organic fertilizers, comprehensive improvement, and non-agricultural land consolidation areas.

### 2.3. Soil Collection and Analysis

#### 2.3.1. Soil Sampling

**Figure 2:**
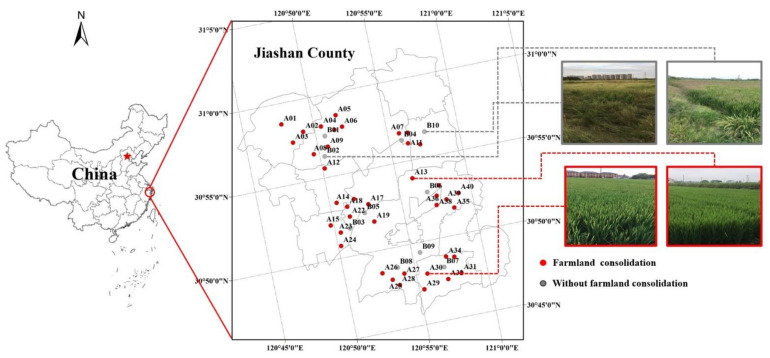
Location map of soil samples.

In September 2018, 40 farmland consolidation areas were randomly selected in the study area (Figure 2). All targeted sites have a rice planting history from 4 to 8 years, after farmland consolidation, numbered A01–A40. Ten plots of cultivated land without farmland consolidation history were randomly selected around the farmland consolidation area. These sites had decades of rice planting history, numbered B01–B10. The farmland in the study area were dominated by green-purple mud fields and yellow-spot fields. The sampling points in the non-agricultural land consolidation area were next to the farmland consolidation area, and had similar soil properties to the farmland consolidation area, which could be used as an effective control group versus the farmland consolidation area. The specific sampling point distribution is shown in Supplementary S1.

At each site, we divided a plot of 10 × 10 m from the area away from the road. We collected surface soil (0–15 cm) samples using five-point method and mixed into one soil sample at each plot [6,38]. Soil samples from all 50 sampling locations were filtered in sterilized stainless steel vessels to remove tree roots, rocks, plant, and animal debris with tweezers. We weighted about 20 g from each soil sample and stored them in a refrigerator at −80 °C for DNA extraction [39]. Five hundred grams of soil samples were stored in a sealed bag and transported back to the laboratory for soil characteristics and heavy metal analysis. After homogeneity, we extracted soil DNA 3 times from each sample and for further analysis.

#### 2.3.2. Soil Basic Physical and Chemical Properties Test

We measured soil pH in the soil solution at 1:2.5 (soil:water) with a digital pH meter. The soil water content (SW) was calculated by using the oven-drying method at 105 °C for 12 h [40]. Soil organic matter (OM) was calculated by measuring the total organic carbon content using a total organic carbon analyzer (BOCS301, Shimadzu, Kyoto, Japan). Soil total nitrogen (TN), soil available phosphorus (AP), soil total phosphorus (TP), and soil available potassium (AK) were respectively determined by automatic Kjeldahl nitrogen analyzer (K9860, Hai Energy, Qingdao, China), flame photometer and spectrophotometer photometric measurement [41]. Soil catalase, urease, and phosphatase activities were determined by sodium phenate, sodium phenol-sodium hypochlorite colorimetry, and phenyl disodium phosphate colorimetry [42]. Each measurement was repeated at least three times for each sample. All data are detailed in Supplementary S2.

#### 2.3.3. Soil Heavy Metal Content Test

We extracted soil samples using HCl-HNO_3_-HF-HClO_4_, the concentrations of Cu, Zn, Cr, Cd, Pb, and Ni in the soil was measured by an inductively coupled plasma source mass spectrometer (Agilent 7800, Palo Alto, CA, USA) [43]. Specifically, we digested 0.1 g soil samples using 3 mL 37% HCl, 1 mL 65% HNO_3_, 6 mL 65% HF, and 0.5 mL 65% HClO_4_. The digestion solution was evaporated to near dryness and dissolved in 1.0 mL of 65% HNO_3_, and then 20 mL of deionized water was added. The concentrations of Hg and As in the soil was pretreated with aqua regia according to the China National Standard (GB 22105-2008) and then measured by an atomic fluorescence spectrophotometer (AF-630, BFRL, Beijing, China) [43]. In this experiment, we repeated measurement 3 times, and used a blank control to ensure the measurement quality.

#### 2.3.4. Soil Microbial Properties Determination

(1) Soil microbial biomass determination

Soil microbial biomass usually includes soil microbial biomass carbon (MBC), microbial biomass nitrogen (MBN), microbial biomass phosphorus (MBP), etc., and it is mainly determined by chloroform fumigation extraction method [44]. We fumigated 10 g of sieved soil and the blank control group with de-ethanol chloroform, added K_2_SO_4_ for shaking extraction and filtered after 24 h. For microbial biomass carbon, after adding K_2_Cr_2_O_7_ and H_2_SO_4_ solution to the extract to boil, FeSO_4_·7H_2_O was added and the coefficient conversion titrated. The microbial biomass nitrogen was processed by adding CuSO_4_ and concentrated H_2_SO_4_ to the extract, then adding NaOH and connecting a distillation nitrogen analyzer to absorb the released NH_3_, and the result was calculated by coefficient conversion. The microbial biomass phosphorus was measured in a spectrophotometer by using NaHCO_3_ and KH_2_PO_4_.

(2) DNA extraction and sequencing analysis

According to the instructions of the FastDNA SPIN kit (MP Biomedicals, Santa Ana, CA, USA), the microbial DNA for PCR amplification was extracted from 0.5 g of soil sample. The Nanodrop 2000 spectrophotometer (Thermo Scientific, Waltham, MA, USA) was used to measure the concentration and quality of the extracted DNA, and the extracted DNA was stored in a refrigerator at −20 °C for later analysis. For bacteria, primers 338F and 806R were used for PCR amplification of the V3–V4 region of the 16S rDNA gene [45].

PCR used 20 µL reaction system, including MdNTPs, FastPfu Buffer, FastPfu Polymerase, primers, BSA, DNA template, and finally added pure water to 20 µL. We carried out the following amplification procedure with ABI GeneAmp^®^ 9700 PCR machine: pre-denaturation at 95 °C for 3 min; 95 °C for 30 s, 60 °C for 30 s, 72 °C for 45 s, repeat 10 cycles; 72 °C extend down for 10 min; perform 30 min at 10 °C. After the amplified products were electrophoresed on a 2% agarose gel, the PCR mixed products were recovered with a gel extraction kit (Omega, Norcross, GA, USA).

We used the Illumina HiSeq4000 platform for sequencing and microbial community analysis [11]. The sequencing data was analyzed on Illumina HiSeq4000, and the original DNA sequencing data was processed by the Quantitative Insights Into Microbial Ecology 2 platform (QIIME 2, University of California, San Diego, CA, USA) [46]. We used Usearch 7.1 to divide high-quality sequences with 97% similarity into operational taxonomic units (OTUs). Before clustering the sequences in OTUs, sequences that only appear once were removed to improve the accuracy of diversity assessment.

### 2.4. Statistic Analysis

The microbial α diversity index could reflect the richness and diversity of microbial communities, including Shannon, Simpsoneven, Simpson and Chao 1, which were all evaluated by the mothur software package (version v.1.30.1, University of Michigan, Ann Arbor, MI, USA) [47]. The composition of the microbial community was mainly displayed in the form of bar graphs and Heatmap graphs through the “vegan” package. The β diversity index was calculated by QIIME to analyze the differences of microbial communities. The drivers of the differences in soil microbial community composition could be processed and discriminated by PLS-DA (Partial Least Squares Discriminant Analysis). The correlations between environmental factors and soil microbial species were analyzed by Spearman method and displayed in the form of Heatmap. The functional composition of soil microorganisms was used to predict the function of amplicon sequencing data of bacteria through PICRUSt2 software (Harvard University, Cambridge, Massachusetts, USA). These were the scientific and effective bioinformatics statistical analysis methods commonly used in the world [11].

We used geological accumulation index (*GI*), proposed by Muller, to assess heavy metal pollution levels, which had been widely used in paddy soils [48]. The Nerome Comprehensive Index (*NI*) is another common method based on *GI* to reflect the level of heavy metal pollution [49]. Collectively, the level of heavy metal pollution in the soil can be effectively assessed by combining the two methods of *GI* and *NI*. *GI* is a geochemical standard for determining the pollution level of a single heavy metal in the soil by comparing it with the pre-industrial level. The formula is as follows:

(1) $$GI_{i}=log_{2}\left(\frac{C_{i}}{1.5B_{i}}\right)$$

where *C_i_* represents the concentration of a single heavy metal in the soil collected from sample *i*; *B_i_* represents the geochemical background value of a single heavy metal in the sample in this area [50]. In addition, one study reported the influence of the external environment and human activities on the fluctuation of metal content can be expressed by a coefficient of 1.5 [51]. *NI* was determined based on the results of *GI*, and a reasonable and comprehensive assessment of pollution could be obtained. The formula is as follows:

(2) $$NI_{i}=\sqrt{\frac{GI_{i_{ave}}^{2}+GI_{i_{max}}^{2}}{2}}$$

where *GI_iave_* and *GI_imax_* show the average and maximum *GI_i_* values of eight heavy metals, respectively. According to the *NI* value, the level of heavy metal pollution can be divided into severe level (SL, *NI* > 3), moderate level (ML, 2 < *NI* ≤ 3), light level (LL, 1 < *NI* ≤ 2), and clean level (CL, *NI* ≤ 1) [52].

## 3. Results and Discussion

### 3.1. Effect of Land Consolidation on Soil Bacterial Community

#### 3.1.1. Changes in Soil Bacterial Diversity

Soil microorganisms include bacteria, fungi, archaea, actinomycetes, viruses, algae, and protozoa. Among them, bacteria have an absolute advantage in quantity and volume play an important role in the operation of soil ecosystems [53,54]. Therefore, this article selected bacteria to represent soil microorganisms for analysis to determine the degree of impact of different farmland consolidation measures on soil microbial diversity. α diversity can characterize community diversity characteristics such as the number of species, uniformity, and relative abundance of microbial communities in the habitat, while β diversity can reflect the differences between different groups of microbial communities to analyze changes in space. These two microbial diversity indexes can reveal the structure and stability of microbial ecosystem functions, which have been widely used for diversity analysis.

##### Analysis of α Diversity of Bacterial Community

**Figure 3:**
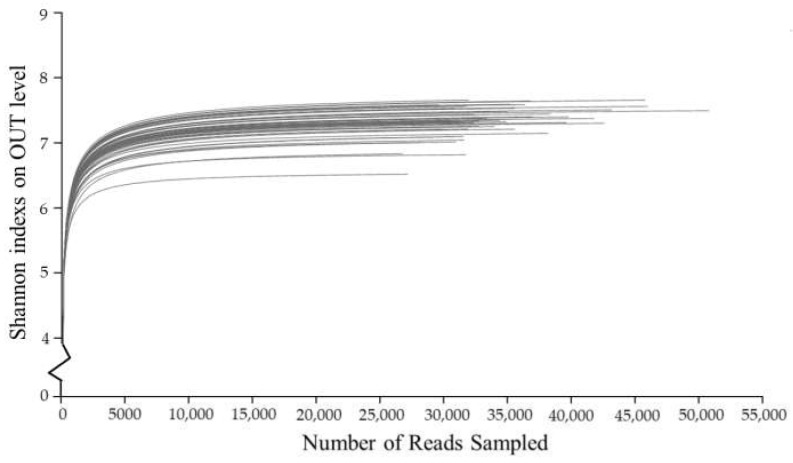
Shannon dilution curve of different samples.

After 16S rDNA amplification and quality control optimization of soil samples in the study area, a total of 1,249,000 valid sequences were obtained, with an average length of about 439 bp, and aggregated into 11,295 OTUs. By making the Shannon dilution curve (Figure 3), a curve that tends to be flat was obtained. This showed that the amount of sequencing data in this experiment was appropriate and sufficient to reflect most of the bacterial diversity information in the soil sample. It was impossible to generate more strains if the amount of sequencing data continues to increase. Therefore, the 16S rDNA sequencing data obtained in this experiment had been able to accurately reflect the needs of bacterial diversity.

After calculating the α diversity index of bacterial communities in soil samples, the correlation and significant relationship between the α diversity indexes of different groups of soil samples were calculated through Spearman correlation analysis (Table 1). The α diversity index mainly included Sobs and Chao reflecting the community richness index, Simpsoneven and Shannoneven reflecting the community evenness index, Shannon and Invsimpson reflecting the community diversity index, and Coverage reflecting the community coverage. The community richness index refers to the sum of the abundance of all microbial species in the soil sample. The larger the value, the richer the community species. In the study area, the soil bacterial community richness indexes Sobs and Chao of the farmland consolidation were significantly higher than those in the non-agricultural land consolidation area at the *p* < 0.05 level. Simpsoneven and Shannoneven, reflecting the community evenness index, could be used to measure the consistency of the relative abundance of microbial community species. This species index did not show the significant difference among the groups, but the value of farmland consolidation areas was higher than that in non-agricultural land consolidation areas. The results indicated, after farmland consolidation, the bacterial community species abundance in cultivated soil will be more uniform. The community diversity index Shannon and Invsimpson were widely used indexes to reflect the community diversity. The higher the value, the higher the community diversity. In the study area, the soil bacterial community diversity index in the farmland consolidation area was significantly higher than that in the non-agricultural land consolidation area, especially in the comprehensive consolidation area, the soil bacterial community diversity index was the highest. The community coverage index could reflect the depth of sequencing, and the value close to 1 indicating that the depth of sequencing was sufficient to cover most of the microbial community species in the soil sample. The Coverage index of soil bacteria in the study area was all greater than 0.95, indicating that the experimental data had high credibility and operability.

Our results showed that the analysis of Shannon dilution curve and Coverage index indicated that the data of this experiment was of high quality and strong operability. The measurement and calculation of the bacterial community α diversity index confirmed that farmland consolidation had a stronger effect on improving the diversity of soil bacterial communities, which was mainly reflected in the community richness index and community diversity index.

##### Analysis of β Diversity of Bacterial Community

β diversity analysis is usually used to compare and analyze the diversity of microbial communities between different sample groups, that is, the difference analysis between samples, mainly by evaluating the abundance information of community species and the evolutionary relationship between samples to measure the distance between samples—so as to effectively reflect the significant differences in microbial communities between different sample groups. PCoA analysis is a commonly used method for β diversity analysis. The R language is used to sort the eigenvectors and eigenvalues, and the most important eigenvalues are selected for representation in the coordinate system. Identify the principal components that have an important influence on the composition of the sample microbial community by way of dimensionality reduction.

**Figure 4:**
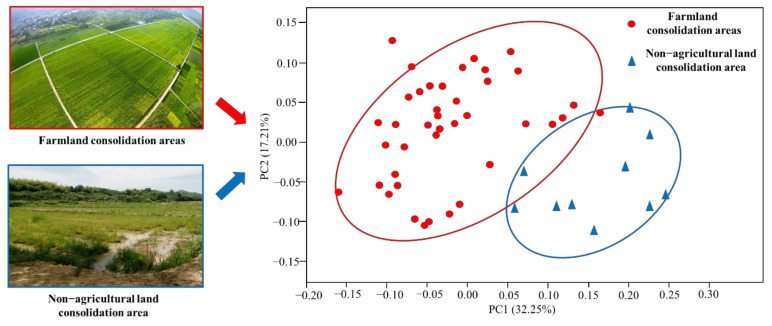
PCoA diagrams of bacterial communities with OTU levels of samples between different groups.

In this study, all samples were divided into two groups: farmland consolidation area and non-agricultural land consolidation area for PCoA analysis. Our results showed that the bacterial communities in the two areas had different clusters, which indicates that there were obvious differences in the soil bacterial community structure between the groups (Figure 4). Two components explained more than 50% for the difference in bacterial community composition of the samples. Hence, we concluded that farmland consolidation affects the change of bacterial community structure by changing the physical and chemical properties of the soil.

#### 3.1.2. Variations in Soil Bacterial Community Structure

##### Analysis on the Changes of Bacterial Community at the Phylum Level

**Figure 5:**
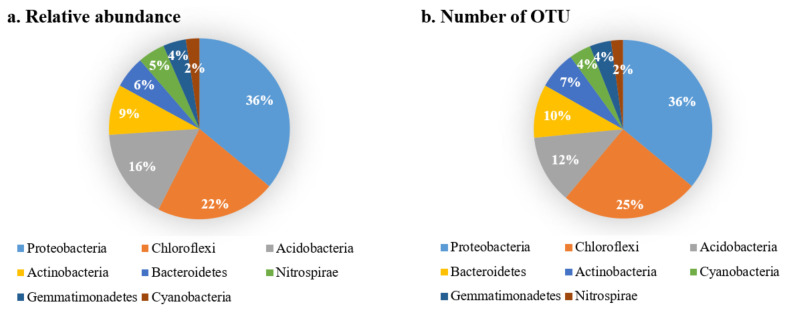
The relative abundance (**a**) and OTU (**b**)composition of the dominant bacteria in the soil samples in the study area.

A total 1,249,000 high-quality soil bacterial sequences were obtained from 50 samples in this study, which were aggregated into 11,295 bacterial OTUs. These high-quality sequences could be divided into 56 bacterial phyla. The dominant phyla with relative abundance greater than 2% included Proteobacteria, Chloroflexi, Acidobacteria, Actinomycetes, Actinobacteria, Bacteroidetes, Nitrospirae, Gemmatimonadetes, Cyanobacteria. The number of effective sequences of these dominant bacteria phyla was 1,112,282, accounting for about 89% of the total; the number of OTUs is 7861, accounting for about 70% of the total. Among them, the relative abundance was absolutely dominant in three phyla. Among them, the sequence number and OTU number of Proteobacteria were 399,929 and 2830 respectively, accounting for about 32% and 25% respectively. The sequence number and OTU number of Chloroflexi were 239,428 and 1972, respectively, accounting for 19% and 17%, respectively. The sequence number and OTU number of Acidobacteria were 182,952 and 973, respectively, accounting for about 15% and 9% respectively. The sequence numbers and OTU numbers of Actinobacteria were 100,050 and 541, respectively, accounting for about 8% and 5%, respectively. The sequence numbers and OTU numbers of Bacteroidetes were 63,609 and 760, respectively, accounting for about 5% and 7%, respectively. The sequence numbers and OTU numbers of Nitrospirae were 53,756 and 170, respectively, accounting for about 4% and 2%, respectively. The sequence numbers and OTU numbers of Gemmatimonadetes were 45,718 and 305, respectively, accounting for about 4% and 3%, respectively. The sequence numbers and OTU numbers of Cyanobacteria were 26,840 and 310, respectively, accounting for approximately 2% and 3%, respectively (Figure 5).

**Figure 6:**
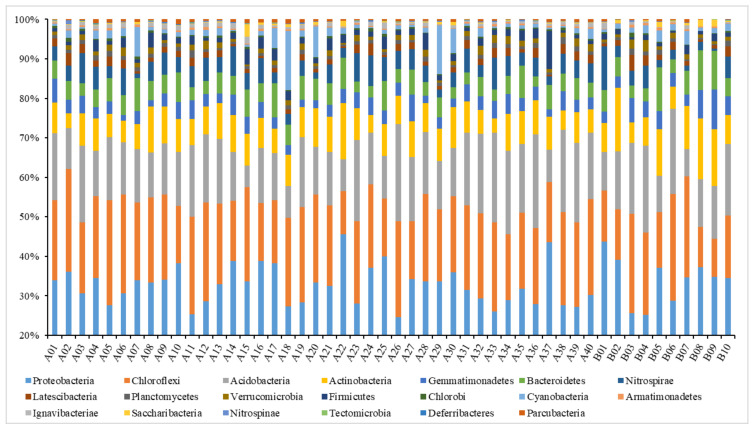
Relative abundance of dominant bacterial phyla in all samples of the study area.

The relative abundance of the soil samples in the top 20 dominant bacterial phyla was extracted for comparison (Figure 6). The abscissa in the Figure 6 represented the sample number, which was introduced in Supplementary S1. The results showed that the average relative abundance of Chloroflexi, Gemmatimonadetes, and Saccharibacteria in all grouped soils in the farmland consolidation area was higher than that in the non-agricultural land consolidation area. However, the average relative abundance of Bacteroidetes, Nitrospirae, Firmicutes, Ignavibacteriae, Spirochaetae, and Nitrospinae was lower than that of non-agricultural land consolidation area. Our results indicated that the farmland consolidation in the study area could increase the relative abundance of the three dominant bacteria phyla, but it also had a negative impact on the relative abundance of the six dominant bacteria phyla.

##### Analysis on the Changes of Bacterial Community at the Genus Level

**Figure 7:**
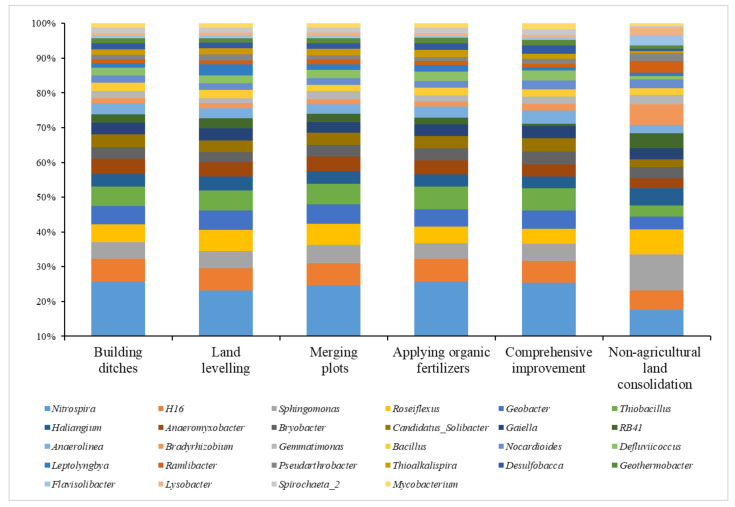
Relative abundance of dominant bacterial genera in all soil samples in the study area.

We divided the sampling site type into six groups: building ditches, merging plots, land levelling, applying organic fertilizers, comprehensive improvement, and non-agricultural land consolidation areas, and extracted the top 20 dominant bacterial genera with relative abundance in each group of soil samples for comparative analysis (Figure 7).

A total of 1,249,000 high-quality soil bacterial sequences from 50 samples in the study area could be divided into 1103 bacterial genera. Specifically, the dominant bacterial genera with relative abundance greater than 1% included *Nitrospira, H16, Sphingomonas,* and *Roseiflexus*. The number of effective sequences of these dominant bacteria was 98,949, accounting for about 8% of the total. Among the collected soil samples, the average relative abundance of *Nitrospira* was the highest, about 4.30%, which was significantly higher than that of other bacterial genera. This genus accounted for the largest proportion of 4.97% of the soil in the plots where comprehensive improvement measures were implemented, and the lowest proportion of soil in the non-agricultural land consolidation area was 3.66%. The average relative abundance of *H16* is 1.23%, which was relatively close in all soil groups, and its proportion ranges from 1.16 to 1.25%. The average relative abundance of *Sphingomonas* was 1.21%, the largest proportion of soils in non-agricultural land consolidation areas was 2.14%, and the lowest proportion in the soil with applying organic fertilizers was 0.84%. The average relative abundance of *Roseiflexus* was 1.18%, the largest proportion of soils in non-agricultural land consolidation areas was 1.52%, and the lowest proportion of soils under comprehensive improvement was 0.82%.

##### Analysis of Changes in Bacterial Community Function

**Figure 8:**
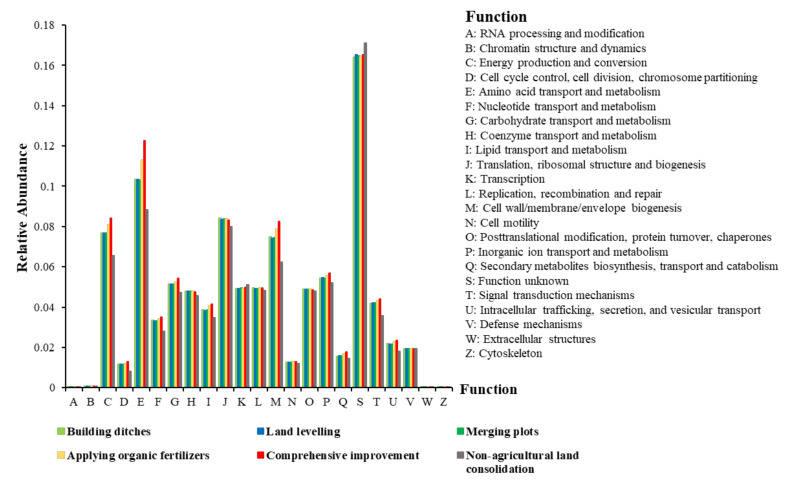
The relative abundance of bacterial community functions in soil samples between different groups.

In order to analyze the differences in soil bacterial functions between different groups, COG family information was obtained through the PICRUSt2 software to calculate the corresponding functional abundance and perform a significant analysis (Figure 8). According to the prediction results of soil bacterial community function, the most important functions included energy production and conversion, amino acid transport and metabolism, ribosome structure and biogenesis, and cell wall/membrane biogenesis. The relative abundance of bacterial community functions such as energy production and transformation, amino acid transport and metabolism, cell wall/membrane biogenesis in the soil of farmland consolidation areas was significantly higher than that in non-agricultural land consolidation areas (*p* < 0.01). This was caused by the significant differences in the composition and structure of the corresponding bacterial communities.

### 3.2. Farmland Consolidation Regulates the Basic Physical and Chemical Properties of Soil and Its Mechanism of Action on Bacteria

#### 3.2.1. Farmland Consolidation Promotes Changes in Basic Physical and Chemical Properties of Soil

##### Soil Physical Properties

The targeted soil physical properties in this study consisted of soil particle size and soil water content, which were the core content of current soil physics research. Soil particle size can directly affect the distribution of soil pores and have a significant impact on the aeration and water holding capacity of the soil. A reasonable particle size distribution is conducive to the healthy growth of crop roots and effectively increases food production [55]. Soil moisture is an important driver for soil physical processes, which can be affected by natural factors such as vegetation, terrain, climate, and human activities at different scales [56], but it can also affect the nutrient cycles and energy flow in the soil environment and directly affect the growth and development of crops.

**Figure 9:**
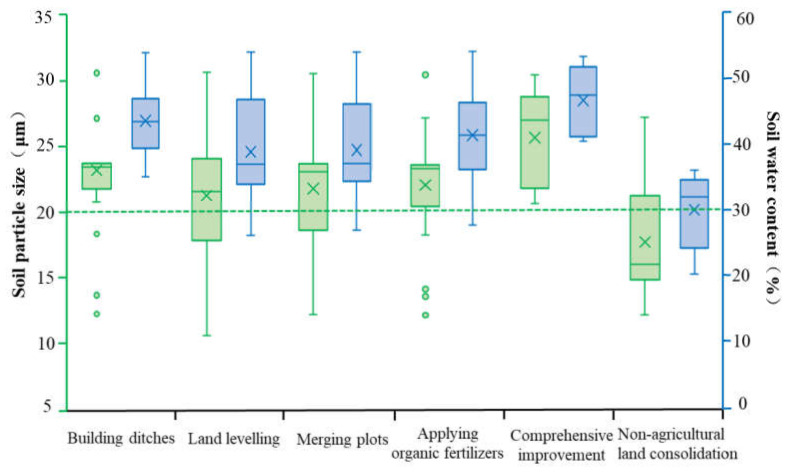
Distribution of soil particle size (green) and soil water content (blue) in the study area.

The soil particle size in the study area ranged from 10.16 to 30.42 μm, and the average soil particle size in the non-agricultural land consolidation area was the smallest, which was significantly lower than that in the farmland consolidation area (Figure 9). Studies had shown that when the soil particle size in paddy soil was less than 20 μm, the content of soil organic matter could be significantly reduced, and when the soil particle size ranged from 20 to 250 μm, it would play a good carbon sink function [57,58]. This was probably because soil particles with a larger particle size have better permeability, the positive charge carried by themselves was easy to combine with the humus carrying negative charges, and it could be decomposed by soil microorganisms easily to increase the organic matter content in the soil [59]. In this study area, the average soil particle size in each farmland consolidation grouping was greater than 20 μm and the distribution was relatively uniform, while the average soil particle size in non-agricultural land consolidation was only 17.48 μm. Our results suggested that farmland consolidation could change soil particle size distribution through measures such as project implementation and soil fertilization, which had a certain driving effect on the change of soil properties. The soil water content in the study area ranged from 20.70% to 54.18%. The comprehensive improvement area had the highest average soil water content (47.00%), followed by the average soil water content of the plots where building ditches were implemented (43.87%). The average soil water content in non-agricultural land consolidation areas was the lowest (30.39%). Our results indicated that the improvement of farmland irrigation facilities and the optimization of soil mechanical structure could effectively increase soil water content and provide sufficient water supply for crop growth.

##### Soil Chemistry

Soil chemistry is an important branch of soil science, including soil pH, organic matter, available potassium, available phosphorus, and other indicators. It plays a key role in the process of soil productivity, self-purification capacity, carbon emissions, and nutrient balance [60]. The current frontier research field of soil chemistry is a cross-discipline of soil chemistry and microbiology. Soil microbes play an important role in the development of soil chemistry research.

The soil pH value in the study area ranged from 6.15 to 8.30. The average soil pH value in the non-agricultural land consolidation area was the highest and the value fluctuates the most, which was significantly higher than areas where the soil pH was close to neutral, such as building ditches, merging plots, applying organic fertilizers, and comprehensive improvement (Table 2). This was because measures such as building ditches and merging plots could effectively promote the flow of water in the farmland, while applying organic fertilizers could reduce the input of inorganic fertilizers, which could effectively regulate the pH of the soil [61]. For soil nutrients, the content ranges of organic matter, available phosphorus, available potassium, and total nitrogen are 16.00~70.40 g/kg, 8.56~330.79 mg/kg, 14.45~80.25 μg/mL and 0.93~3.75 g/kg, respectively. The average soil nutrient of non-agricultural land consolidation areas was the lowest, and was significantly lower than that of farmland consolidation areas, especially the areas where organic fertilizers and comprehensive improvement were applied have higher soil nutrient content. Our results showed that the application of organic fertilizers in farmland consolidation areas could effectively improve soil nutrients, and the construction of ditches accelerates the flow of water to promote nutrient cycling, thereby creating a healthy soil environment to accelerate the promotion of microorganisms in soil nutrient cycling, and achieve benign cycle.

##### Soil Enzyme Activity

Soil enzymes are an important driving factor for soil biogeochemical cycles. They can not only sense changes in soil properties, but their activity represents the capacity of soil nutrient supply, which is a key indicator of soil quality [62]. Among them, catalase is mainly involved in the chemical process of soil redox, which can effectively characterize the content of soil organic matter and the degree of soil decay [63]. Phosphatase and urease participate in the soil nitrogen and phosphorus cycles respectively, and are important indicators to characterize the conversion capacity of soil nitrogen and phosphorus [64]. Additionally, soil enzyme activities can also interact with soil microbial communities. For example, soil enzymes can participate in the degradation of microbial residues, and changes in the structure of microbial communities will also affect soil enzyme activities, thereby affecting the process of soil organic matter decomposition and nutrient cycling [65]. In addition, fertilization and engineering measures will also have a certain impact on soil enzyme activity. Studies have shown that applying nitrogen fertilizer and increasing soil moisture can significantly increase soil enzyme activity [66,67]. Therefore, this study selected catalase, phosphatase, and urease for determination and comparison between groups to try to analyze the effect of farmland improvement on soil enzymes.

According to the results of laboratory tests, in the study area, the soil catalase activity ranged from 125.45 to 276.90 mg/g, and there was no significant difference between the groups (Table 3). However, the soil catalase activity in the non-agricultural land consolidation area was the lowest, with an average of 183.58 mg/g. The soil phosphatase activity ranged from 2.80 to 45.20 mg/g. Among them, the soil phosphatase activity of comprehensive improvement was the highest, and the soil phosphatase activity of farmland consolidation areas was significantly higher than that of non-agricultural land consolidation areas. The soil urease activity ranged from 0.01 to 0.98 mg/g, and there was no significant difference between the groups. However, the soil urease activity in the non-agricultural land consolidation area was the lowest, and the soil urease activity in the comprehensive improvement was the highest. Farmland consolidation had effectively improved the soil environment of farmland by adjusting soil pH, improving soil nutrients, accelerating soil water circulation, and had a greater promotion effect on the increase of soil enzyme activity [68,69].

#### 3.2.2. The Mechanism of Basic Physical and Chemical Properties of Soil on Bacterial Community

##### Soil Physical Properties and Bacterial Community

Soil particle size (SPD) and soil water content (SW) are important indicators for evaluating soil physical properties. They have a strong influence on other soil physical and chemical properties and microbial characteristics, and can directly or indirectly affect crop growth and food quality [70]. Soil particle size is an effective index that can characterize soil porosity and aeration. The size of soil particle size determines the amount of humus that it adheres to, and also reflects the ability to provide nutrients for microorganisms. The soil water content can reflect the water supply capacity and nutrient retention capacity of the soil. As the soil water content increases, it will promote the reproduction of soil microorganisms to increase the number and relative abundance of microorganisms [71]. Therefore, soil particle size and soil water content are key indicators of soil physical properties that affect the structure and diversity of soil microbial communities. However, soil particle size and soil water content are easily affected by farmland consolidation projects, so the correlation analysis of soil particle size, water content, and soil microorganisms in farmland consolidation areas has certain research significance.

**Figure 10:**
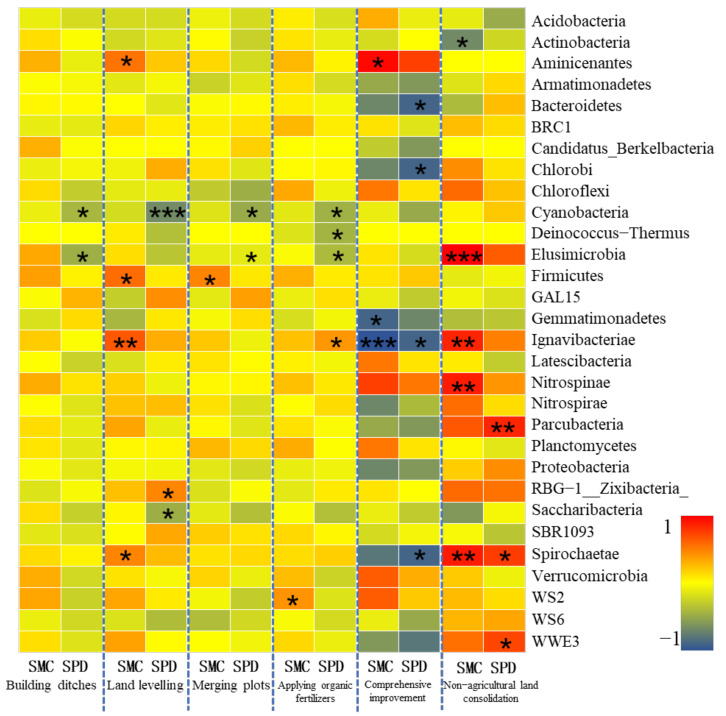
Correlation between soil particle size, moisture content and bacterial community structure in different groups. (Note: Warm colors indicate positive correlation, and cool colors indicate negative correlation. *, **, *** indicate significant correlation at the levels of 0.05, 0.01, and 0.001, respectively).

According to Spearman correlation measurement, the soil particle size in the study area was significantly correlated with the relative abundance of 11 bacterial phyla, and the soil water content was significantly correlated with the relative abundance of 7 bacterial phyla (Figure 10). In farmland consolidation areas where construction of ditches was implemented, the soil water content and the relative abundance of bacteria were mainly positively correlated, but not significant. This was probably because the soil water content of all plots was higher after the construction of ditches in the farmland, so there was no significant difference. The soil particle size in this type of farmland was significantly negatively correlated with Cyanobacteria and Elusimicrobia. This was because these two bacteria tend to be synergetic in a eutrophic environment. However, the area with larger soil particle size would accelerate the water cycle and inhibit the growth of the relative abundance of these two bacteria. In farmland consolidation areas where land levelling was implemented, the soil water content was significantly positively correlated with the relative abundance of Aminicenantes, Firmicutes, Ignavibacteriae, and Spirochaetae. This was because the farmland soil that implemented the land levelling was subjected to certain mechanical compaction, which would affect the physical characteristics of the soil’s water circulation and air permeability, which led to the increase in the relative abundance of some bacteria phyla with the increase of soil water content. However, the Cyanobacteria and the soil particle size were significantly negatively correlated at the level of 0.001. This was due to the mechanical compaction of the soil particle morphology, which resulted in the deterioration of soil voids and water ventilation performance, which restricted the reproduction of the cyanobacteria, and the larger the soil particle size, the greater the impact it would bear. In farmland where combined plots, application of organic fertilizers, and comprehensive improvement were implemented, soil water content and particle size affected the relative abundance of Cyanobacteria, Firmicutes, Elusimicrobia, and Ignavibacteriae, which were probably due to the combined effects of soil nutrients, pH and other environmental factors, and had a certain universality. Different from farmland consolidation areas, the influence of soil water content and particle size on the relative abundance of bacterial communities in non-agricultural land consolidation areas was mostly significantly positively correlated. This was probably due to the uneven drainage and nutrient supply in the non-agricultural land consolidation area. The bacterial community could obtain a better living environment in the soil with high water content and large particle size, thereby significantly increasing its relative abundance. These conclusions were consistent with the previous studies [72].

##### Soil Chemical Properties and Bacterial Communities

Soil pH, organic matter (SOM), available phosphorus (AP), available potassium (AK), and total nitrogen (TN) are representative indicators of soil chemical properties, which can effectively characterize the basic chemical properties and fertility status of the soil, and are also important factors affecting the structure of bacterial communities [73]. Previous studies have shown that the pH value in the black soil is the primary influencing factor that affects the bacterial community structure, while the total nitrogen in the soil under continuous soybean cropping conditions is a key environmental factor that affects soil bacteria [74,75]. Because bacterial communities are more sensitive to changes in soil nutrients and pH [76], and farmland consolidation measures have a strong influence on soil chemical properties. Therefore, the analysis of the correlation between soil chemical properties and bacterial communities in farmland consolidation areas has a certain reference value for the research on farmland microecology.

**Figure 11:**
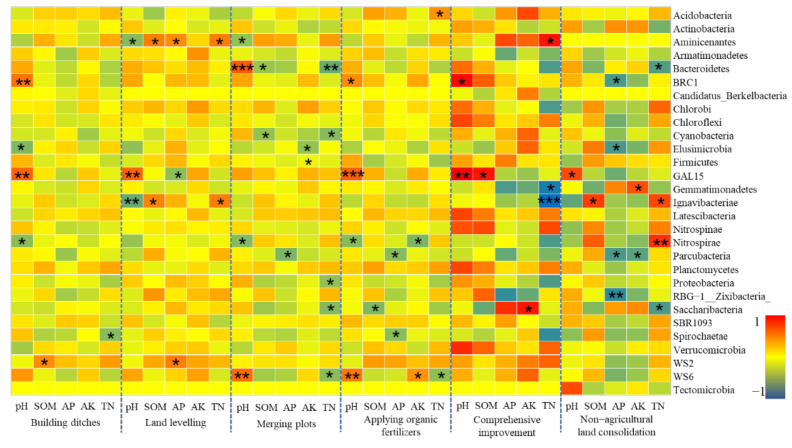
Correlation between soil chemical properties and bacterial community structure in different groups. (Note: Warm colors indicate positive correlation, and cool colors indicate negative correlation. *, **, *** indicate significant correlation at the levels of 0.05, 0.01, and 0.001, respectively).

Spearman correlation analysis was carried out on the relative abundance of bacterial communities and soil chemical properties in different groups of farmland in the study area. The results showed that a total of 18 bacterial phyla were significantly correlated with soil chemical indicators such as soil pH, SOM, AP, AK, and TN (Figure 11). The 8 bacterial phyla that were significantly related to pH include Bacteroidetes, Aminicenantes, Ignavibacteriae, and Nitrospirae, which mainly appeared in farmland consolidation areas. This was probably because the engineering measures in the farmland consolidation area had a certain adjustment effect on the soil pH, but they also interfered with the soil environment, resulting in uneven pH distribution and a significant impact on the relative abundance of soil bacteria in local plots. There were 7 bacterial phyla that were significantly affected by SOM, and the relative abundance of soil bacteria in the plots where comprehensive improvement was implemented showed a strong positive correlation with SOM, including Latescibacteria, Nitrospinae, Planctomycetes, and other phyla. Our results suggested that increasing soil organic matter content and improving nutrient cycling conditions through farmland consolidation had a greater promotion effect on the survival and reproduction of soil bacteria. AP, AK, and TN, as important soil nutrients, also had a greater impact on the structural changes of soil bacteria. A total of 17 bacterial phyla were significantly related to these three nutrients. Among them, there were 9 bacterial phyla in non-agricultural land consolidation areas that were significantly correlated with soil AP, AK, and TN, but most were negatively correlated. Our results indicated that, due to the massive application of inorganic fertilizers in non-agricultural land consolidation areas, the soil environment was polluted and soil nutrient dynamic balance was destroyed, thereby reducing soil bacterial activity [77].

##### Soil Enzyme Activity and Bacterial Community

Soil microbes and crop secretions are important sources of soil enzymes, so soil enzyme activity can be used as an indicator of soil microbial activity and has a close relationship with the structure of soil bacterial community [78]. Existing studies have shown that soil urease (URE), phosphatase (PDXP), and catalase (CAT) were significantly positively correlated with the total amount of soil bacteria (*p* < 0.05) [79]. There are also many studies on the relationship between soil enzyme activity and bacterial community structure, but they only stay at the level of bacterial diversity analysis without performing correlation analysis on specific bacterial species [79,80].

**Figure 12:**
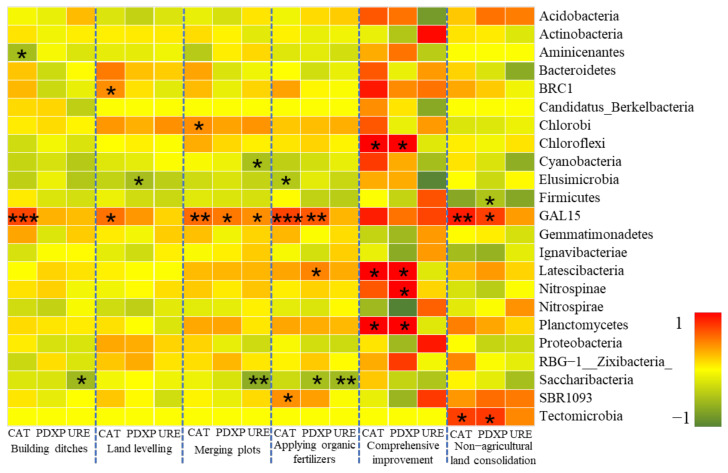
The correlation between soil enzyme activity and bacterial community structure in different groups. (Note: Warm colors indicate positive correlation, and cool colors indicate negative correlation. *, **, *** indicate significant correlation at the levels of 0.05, 0.01, and 0.001, respectively).

A total of 14 bacterial phyla in farmland soil samples in the study area were significantly correlated with soil enzyme activity indicators such as URE, PDXP, and CAT (Figure 12). In the farmland consolidation area, the soil enzymes in the farmland where the organic fertilizer was applied and the comprehensive improvement was carried out had a significant positive correlation with the more dominant bacteria phyla. This was because the two groups of soils had a higher content of soil enzymes, and the bacteria obtained soil nutrients such as carbon, phosphorus, and nitrogen decomposed by enzymes could achieve rapid reproduction. The phylum Chloroflexi and Planctomycetes were significantly positively correlated with catalase (CAT) and phosphatase (PDXP) in farmland soils under comprehensive improvement. This was because many bacteria in these two phyla could produce energy and nutrient elements through photosynthesis and oxidation, which could help the microorganisms and enzyme activities in the soil to a certain extent. However, in farmland soils in non-farmland consolidation areas, most bacterial phyla had a negative correlation with soil enzymes. This was also because there were fewer nutrients available for soil enzyme decomposition in this group, and it was difficult to promote the development of the bacterial community, which was caused by the microecological imbalance.

### 3.3. Farmland Consolidation Regulates Soil Heavy Metal Content and Its Mechanism of Action on Bacteria

#### 3.3.1. Effects of Farmland Consolidation on Soil Heavy Metal Content

##### Heavy Metal Content of Farmland Soil

The levels of heavy metals in farmland soils of different groups in the study area are shown in Table 4. Among the eight heavy metals tested in farmland soils in the study area, the average content of seven heavy metals was greater than the background value of the soil, and only the average content of the heavy metal As was slightly lower than the background value. Among them, the average contents of Cu, Cd, Pb, Cr, Hg, Ni, and Zn were 45.17, 2.5, 41.94, 223.79, 0.55, 56.77, 123.27 mg/kg, respectively, which were 2.00, 14.71, 1.17, 4.00, 3.24, 2.38, 1.48 times of the soil background value in the region. This showed that there was a relatively serious accumulation of heavy metals in farmland soils in this study area.

The order of the coefficient of variation of the eight heavy metals in the study area was: As > Hg > Pb > Cd > Cu > Zn > Ni > Cr. Among them, the coefficients of variation of As, Hg, Pb, Cd, Cu and Zn were all greater than 20%, belonging to moderate intensity variation, indicating that these heavy metals were significantly affected by external interference. The specific manifestation was that the content of heavy metals in the soil varies greatly in space, which was mainly attributed to the influence of human disturbance factors such as farming methods, fertilizer application, management measures, and pollutant emissions. The small coefficient of variation of Cr and Ni indicated that the spatial distribution of these two heavy metal elements was relatively uniform, and there might be a certain degree of homology [81]. As an artificial measure that strongly disturbed the soil environment, farmland consolidation was an important factor affecting the spatial distribution of soil heavy metal content. There were large differences in the content of heavy metals between the areas where different agricultural land consolidation measures were implemented and the non-agricultural land consolidation areas, and the highest average values of various heavy metal content were in the non-agricultural land consolidation areas. The content of heavy metals in farmland where construction of ditches, land levelling, combined plots, application of organic fertilizers, and comprehensive improvement were implemented was lower than that of non-agricultural land consolidation areas. This was probably because the construction of ditches could significantly improve the transfer of heavy metals by water in the soil, and the application of organic fertilizers could provide nutrients for the growth and reproduction of microorganisms to play the function of transferring and absorbing related heavy metals, which was consistent with the existing research results [82].

##### Heavy Metal Pollution Level of Farmland Soil

**Figure 13:**
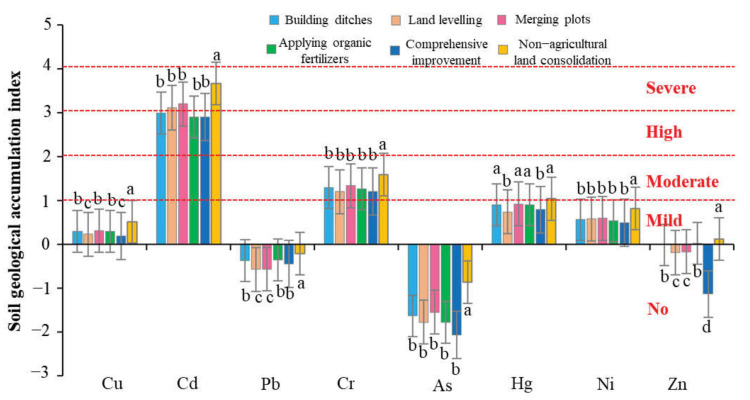
Evaluation results of soil geological accumulation index in the study area. Note: Different lowercase letters represent significant differences at the *p* < 0.05 level.

According to the results of the Geological Accumulation Index (*GI*) (Figure 13), the pollution degree of the heavy metals in the study area was in descending order: Cd > Cr > Hg > Ni > Cu > Zn > Pb > As. Among them, Cd was the heavy metal with the highest geological accumulation index and the most polluted heavy metal in the study area. The geological accumulation index of the three heavy metals Pb, As, and Zn was mostly negative, indicating that the soil in the study area was not polluted by them [83]. At the same time, there were big differences in the geological accumulation level of heavy metals among different groups. In the study area, the cumulative geological index of heavy metals in the farmland under comprehensive improvement was generally low, while the cumulative index of heavy metals in the non-agricultural land consolidation area had the highest value.

**Figure 14:**
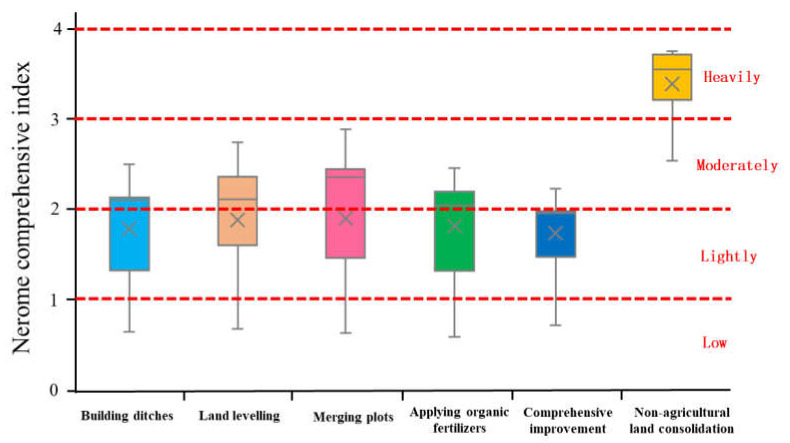
Evaluation results of Nerome comprehensive index in the soil of the study area.

The comprehensive pollution level of heavy metals in farmland soil could be determined by measuring the Nerome comprehensive index (*NI*). The average range of the soil Nemerow comprehensive index of each group in the study area was 0.64 to 3.68. According to the soil heavy metal pollution index classification standard [52], when the *NI* is less than 1, the soil is low pollution; when the *NI* is 1 to 2, the soil is lightly polluted; When the *NI* is 2 to 3, the soil is moderately polluted, which will pose a toxic threat to rice; when the *NI* is 3 to 4, the soil is heavily polluted, which will seriously affect the growth and development of crops. According to the calculation results of Nerome comprehensive index, the farmland soil in the non-agricultural land consolidation area was at a heavily polluted level, the farmland soil in the combined plot was at a moderately polluted level, and the farmland soil in other farmland consolidation areas was at a lightly polluted level (Figure 14). This was because the implementation of construction ditches could promote soil water circulation to improve the effective transfer of heavy metals, and the application of organic fertilizers could increase the abundance of heavy metal-tolerant bacteria to adsorb, migrate and detoxify related heavy metals. All of these could effectively reduce the level of heavy metal pollution in farmland consolidation areas. This conclusion was consistent with the existing research results [82]. At the same time, this also explained why the farmland soil that implements comprehensive improvement had the lowest level of heavy metal pollution.

Through the above calculation analysis and field investigation, our results showed that the reasons for the large differences in the level of heavy metal pollution in farmland soils in the study area were as follows:

(1) In recent years, the area where the study area was located has continuously strengthened the development and utilization of the industrial area, causing industrial pollutants to continue to flow into the surrounding farmland, causing serious soil heavy metal pollution. (2) Many oily pollutants, such as fertilizer bags, pesticide bottles, and herbicides, which were likely to cause cadmium (Cd) pollution, were scattered in the farmland, but had not been effectively removed. (3) Pollutants caused by fertilizer application, poultry breeding and domestic garbage in the study area were also sources of heavy metal pollution in the soil [84]. (4) Measures such as building ditches and applying organic fertilizers in farmland consolidation areas could accelerate water circulation to transfer heavy metals, and provide nutrients for bacteria with heavy metal repair functions to achieve the adsorption and detoxification of heavy metals, which effectively reduced the level of heavy metal pollution in farmland [85].

#### 3.3.2. The Mechanism of Soil Heavy Metal Pollution on Bacterial Communities

##### Bacterial Communities at Low Pollution Levels

**Figure 15:**
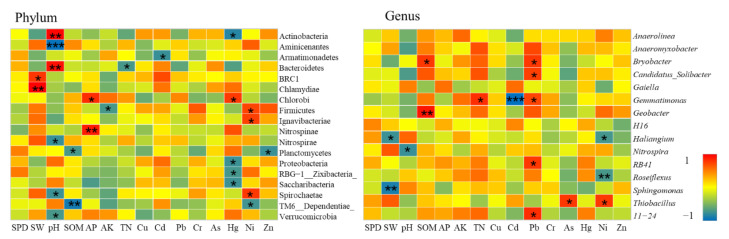
Correlation between soil environmental factors and bacterial community structure with low heavy metal pollution levels. (Note: Warm colors indicate positive correlation, and cool colors indicate negative correlation. *, **, *** indicate significant correlation at the levels of 0.05, 0.01, and 0.001, respectively).

A total of 10 samples in the study area belonged to the low level of heavy metal pollution, all located in the farmland consolidation area. According to CCA analysis, in these 10 samples, heavy metals Cr (r^2^ = 0.30, *p* = 0.027), As (r^2^ = 0.27, *p* = 0.032), Hg (r^2^ = 0.28, *p* = 0.031), Ni (r^2^ = 0.45, *p* = 0.013) were significantly related to bacterial community structure. According to Spearman correlation analysis, the content of heavy metals was significantly related to 11 bacterial phyla and 8 bacterial genera (*p* < 0.05) (Figure 15).

At the bacterial phyla level, the relative abundance of soil bacteria in farmland with low soil pollution levels of heavy metals was significantly related to the physical and chemical properties of the soil. In particular, Actinobacteria, Aminicenantes, Bacteroidetes, Chlamydiae, Nitrospinae, TM6__Dependentiae_were significantly correlated with SW, pH, SOM, and AP at the level of 0.01. The relative abundance of soil bacteria in this area was also significantly related to the content of heavy metals such as Cd, Hg, Ni, Zn, and the relative abundance of 9 bacterial phyla were significantly related to the content of Hg and Ni. Among them, Chlorobi had a significant positive correlation with Hg, while Firmicutes, Ignavibacteriae, and Spirochaetae had a significant positive correlation with Ni. Our results suggested that these four types of bacteria have strong adaptability under low pollution levels of heavy metals, and could absorb and transfer heavy metals Hg and Ni to a certain extent. Studies have shown that pH could further change the microbial community structure by affecting the leaching of heavy metals and adjusting nutrients such as AP and SOM, which was consistent with the results of this research [86].

At the level of bacterial genera, *Bryobacter, Gemmatimonas, Geobacter, Haliangium, Nitrospira, Sphingomonas* were significantly related to the basic physical and chemical properties of soil such as SW, pH, SOM, and TN. The heavy metals Cd, Pb, As, and Ni were the main influencing factors affecting the genus structure of bacteria. Among them, *Bryobacter, Candidatus_Solibacter, Gemmatimonas, RB41, 11–24* had a significant positive correlation with Pb content, while *Thiobacillus* had a significant positive correlation with As and Ni. This showed that these bacteria had a certain tolerance to heavy metals such as Pb, As, and Ni in the low pollution level of heavy metals, and had a strong heavy metal degradation function, which had a great effect on improving the soil environment.

##### Bacterial Communities at Light Pollution Levels

**Figure 16:**
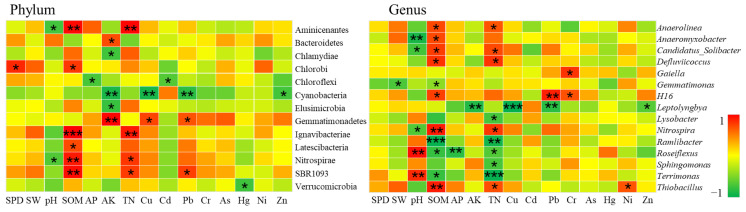
Correlation between soil environmental factors and bacterial community structure at light level of heavy metal pollution. (Note: Warm colors indicate positive correlation, and cool colors indicate negative correlation. *, **, *** indicate significant correlation at the levels of 0.05, 0.01, and 0.001, respectively).

A total of 14 samples in the study area belonged to the level of light heavy metal pollution. One of the samples was located in the non-agricultural land consolidation area, and the rest were in the farmland consolidation area. According to CCA analysis, in these 14 samples, heavy metals Hg (r^2^ = 0.53, *p* = 0.029), Cd (r^2^ = 0.38, *p* = 0.081), Pb (r^2^ = 0.26, *p* = 0.094), As (r^2^ = 0.24, *p* = 0.023), Cr (r^2^ = 0.19, *p* = 0.032) were significantly related to the bacterial community structure. According to Spearman correlation analysis, the content of heavy metals was significantly related to 5 bacterial phyla and 4 bacterial genera (*p* < 0.05) (Figure 16).

At the phylum level, the relative abundance of farmland soil bacteria at this pollution level was significantly related to the physical and chemical properties of soils such as SOM, AK, TN, especially Aminicenantes, Ignavibacteriae, Nitrospirae, and SBR1093. Heavy metals Cu and Pb had a strong influence on the structure of bacterial community, and were significantly positively correlated with Gemmatimonadetes and SBR1093. Our results indicated that Gemmatimonadetes and SBR1093 could still have strong adsorption and degradation effects on heavy metal elements such as Cu and Pb under conditions of sufficient nutrients and in an environment with a light heavy metal pollution level.

At the genus level, the basic physical and chemical properties of soil pH, SOM, TN were important influencing factors that affected the structure of bacterial community, and had a significant impact on the relative abundance of *Candidatus_Solibacter, Nitrospira, Roseiflexus,* and *Terrimonas*. Cu, Pb, Cr, Ni, and Zn were the main heavy metal elements that affected the structure of bacterial genera. Among them, bacterial genera of *Gaiella, H16, Thiobacillus* were significantly positively correlated with the contents of Pb, Cr, and Ni. This showed that these bacterial genera had a certain tolerance to heavy metals such as Pb, Cr, Ni in the light pollution level of heavy metals, and had a strong function of heavy metal degradation, which could effectively improve the soil environmental conditions.

##### Bacterial Communities at Moderate Pollution Levels

**Figure 17:**
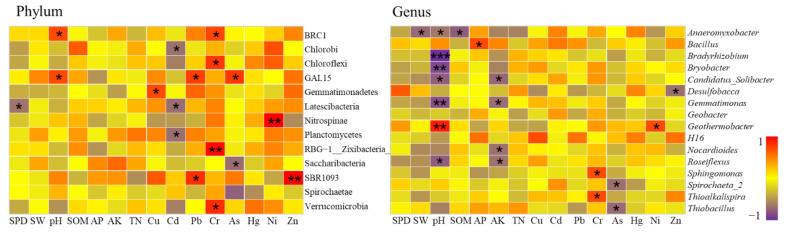
Correlation between soil environmental factors and bacterial community structure at moderate levels of heavy metal pollution. (Note: Warm colors indicate positive correlation, and cool colors indicate negative correlation. *, **, *** indicate significant correlation at the levels of 0.05, 0.01, and 0.001, respectively).

A total of 15 samples in the study area belonged to the moderate level of heavy metal pollution, of which two samples were located in the non-agricultural land consolidation area, and the rest were located in the farmland consolidation area. According to CCA analysis, among these 15 samples, heavy metals Cu (r^2^ = 0.46, *p* = 0.043), Pb (r^2^ = 0.45, *p* = 0.042), Hg (r^2^ = 0.35, *p* = 0.045), Zn(r^2^ = 0.38, *p* = 0.035) were significantly related to bacterial community structure. According to Spearman’s correlation analysis, the content of heavy metals was significantly related to 11 bacterial phyla and 6 bacterial genera (*p* < 0.05) (Figure 17).

At the phylum level, the correlation between the relative abundance of farmland soil bacteria and the basic physical and chemical properties of the soil at the moderate pollution level was not significant, only two phyla were significantly related to pH and one phyla was significantly related to SPD. Cd, Pb, Cr, As were the main heavy metal elements that affected bacteria. Among them, Pb was significantly positively correlated with GAL15 and SBR1093; Cr was significantly positively correlated with BRC1, Chloroflexi, RBG-1__Zixibacteria_, Verrucomicrobia; As was significantly positively correlated with GAL15; and Cd was significantly negatively correlated with Chloroflexi, Latescibacteria, and Planctomycetes. Our results showed that in a moderately polluted environment with heavy metals, the basic physical and chemical properties of soil were no longer the main environmental factors affecting the soil bacterial community. At this pollution level, some of the bacteria still had good tolerance to heavy metals such as Pb, Cr, As, and Cd, and had a strong absorption effect on them.

At the genus level, the soil pH and AK were important influencing factors on the structure of bacterial communities, and had a significant impact on the relative abundance of bacterial genera *Candidatus_Solibacter, Gemmatimonas, Roseiflexus*. The influence of heavy metals on the relative abundance of bacterial genera was limited. Only Cr and Ni had a significant positive correlation with bacterial genera *Sphingomonas, Thioalkalispira, Geothermobacter*. This also showed that under this pollution level, the basic physical and chemical properties of the soil had a greater impact on the community structure of bacterial genera, and some bacterial genera still had a certain degrading effect on heavy metals such as Cr and Ni. This conclusion was consistent with the results of existing literatures [87].

##### Bacterial Communities under Heavy Pollution Levels

**Figure 18:**
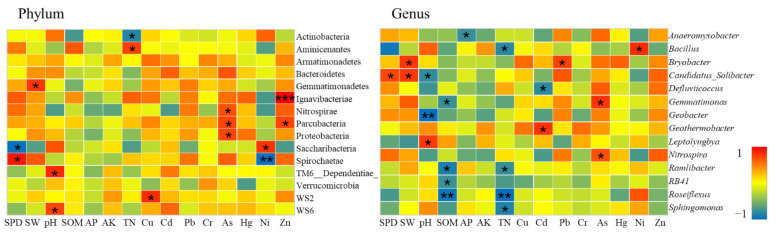
Correlation between soil environmental factors and bacterial community structure at heavy pollution levels. (Note: Warm colors indicate positive correlation, and cool colors indicate negative correlation. *, **, *** indicate significant correlation at the levels of 0.05, 0.01, and 0.001, respectively).

A total of 11 samples in the study area were in the heavily polluted area, of which 7 samples were in the non-agricultural land consolidation area, and the remaining 4 samples were in the farmland consolidation area. According to CCA analysis, in these 11 samples, heavy metals Cd (r^2^ = 0.34, *p* = 0.020), Cr (r^2^ = 0.28, *p* = 0.030), Ni (r^2^ = 0.41, *p* = 0.017) were significantly related to the bacterial community structure. According to the Spearman correlation analysis, the content of heavy metals was significantly related to 7 bacterial phyla, and 6 bacterial genera (*p* < 0.05) (Figure 18).

At the phyla level, the relative abundance of farmland soil bacteria at the heavily polluted level was not significantly correlated with SW, pH, and TN. Only SPD, SW, and pH were significantly related to some bacteria phyla. Cu, As, Ni, Zn were the main heavy metal elements that affect bacteria. Among them, Cu had a significant positive correlation with WS2; As had a significant positive correlation with Nitrospirae, Parcubacteria, Proteobacteria; Ni had a significant positive correlation with Saccharibacteria; and Zn had a significant positive correlation with Ignavibacteriae and Parcubacteria. Our results indicated that there were still a certain number of heavy metal-tolerant bacteria phyla in heavily polluted environment, which played an important role in stabilizing the quality of the soil environment.

At the genus level, nutrient indicators such as SOM and TN had a greater impact on the structure of bacterial community. There was a significant negative correlation with the relative abundance of *Roseiflexus* and *Ramlibacter*. This might be that in this environment, the genus with heavy metal tolerance could significantly inhibit the toxic effects of heavy metals, creating favorable conditions for other microorganisms to absorb nutrients to increase their survival rate, resulting in a rapid decline in nutrients. This was consistent with the research results of Li [88]. The heavy metals Cd and As were the main influencing factors, which had a significant effect on the relative abundance of some bacterial genera. Among them, Cd and *Geothermobacter*, Pb and *Bryobacter*, As and *Gemmatimonas* and *Nitrospira*, Ni and *Bacillus*, all showed a significant positive correlation. Under heavy pollution levels, soil nutrients were particularly important for the survival of microorganisms, and heavy metal-tolerant bacteria provided a more favorable growth environment for other bacteria, which was consistent with existing research conclusions [89].

## 4. Conclusions

This study used soil experimental analysis and high-throughput sequencing technology of gene amplicons to measure the basic physical and chemical properties of soil, soil heavy metal content, and soil microbial characteristics. We used the basic physical and chemical properties of the soil and heavy metal content as an intermediary, to study the impact mechanism of farmland consolidation on microorganisms, to explore the impact of different farmland consolidation measures on the soil micro-ecological environment, and drew the following main conclusions:

(1) Farmland consolidation had a significant impact on soil microbial characteristics, which was mainly manifested in changes in soil microbial biomass, microbial diversity and community structure. The soil microbial biomass carbon and nitrogen in farmland consolidation areas were significantly higher than those in non-agricultural land consolidation areas, and the microbial biomass phosphorus in soil samples from most farmland consolidation areas was significantly higher than that in non-agricultural land consolidation areas. In the study area, the soil bacterial and fungal community richness indexes Sobs and Chao of the cultivated land that had implemented farmland consolidation were significantly higher than the non-agricultural land consolidation areas at the *p* < 0.05 level. The soil bacterial community diversity indexes Shannon and Invsimpson in farmland consolidation areas were significantly higher than those in non-agricultural land consolidation areas, especially the soil bacterial community diversity index in comprehensive improvement areas was the highest.
(2) Farmland consolidation could have a significant impact on the basic physical and chemical properties of the soil. In the study area, the soil particle size and water content of the agricultural land consolidation area were significantly higher than those on the non-agricultural land consolidation area, and the soil pH value of the non-agricultural land consolidation area was significantly higher than that of the construction ditches, combined plots, application of organic fertilizer, and comprehensive improvement areas where the soil pH was close to neutral. Regarding soil nutrients, the content of organic matter, available phosphorus, available potassium, and total nitrogen in non-agricultural land consolidation areas was also significantly lower than that in farmland consolidation areas, especially the application of organic fertilizer and comprehensive improvement areas had higher soil nutrients. In addition, the soil catalase, phosphatase, and urease activities in farmland consolidation areas were significantly higher than those in non-agricultural land consolidation areas. Our results showed that farmland consolidation had effectively improved the soil environment of farmland by adjusting soil pH, improving soil nutrients, accelerating soil water circulation, improving soil enzyme activity, and creating favorable conditions for the survival and reproduction of soil microorganisms.
(3) Farmland consolidation had an indirect impact on soil bacteria by adjusting the basic physical and chemical properties of the soil. Studies have shown that the effects of different farmland consolidation measures on the relative abundance of soil bacteria were quite different. The soil water content in the farmland through the implementation of construction ditches was significantly improved, and the area with larger soil particle size could accelerate the water cycle, thereby effectively inhibiting the increase in the relative abundance of Cyanobacteria and Elusimicrobia. However, in areas with land levelling, Cyanobacteria was significantly negatively correlated with soil particle size. This was due to the mechanical compaction of soil particle morphology, which resulted in soil voids and poor water ventilation performance, which restricted the reproduction of Cyanobacteria. The larger the soil particle size, the greater the impact it would bear. Important soil nutrients such as SOM, AP, AK, TN also had a greater impact on the structural changes of soil bacteria, but there was a significant negative correlation between soil bacteria and soil nutrients in non-agricultural land consolidation areas. This was probably due to the large-scale application of inorganic fertilizers in non-agricultural land consolidation areas, resulting in soil environmental pollution, destroying the dynamic balance of soil nutrients, and reducing soil bacterial activity. In addition, in farmland consolidation areas, there was a significant positive correlation between soil enzymes and more dominant bacteria in farmland where organic fertilizers were applied and comprehensive improvement was implemented. This was because these two groups of soils had a high content of soil enzymes, and bacteria could reproduce quickly by obtaining nutrients such as carbon, phosphorus, and nitrogen that were decomposed by soil enzymes.
(4) Farmland consolidation had a significant effect on the content of heavy metals in the soil. Among the eight heavy metals tested in the farmland soil of the study area, the average content of seven heavy metals was greater than the background value of the soil, and only the average value of As was slightly lower than the background value. This showed that there was a relatively serious accumulation of heavy metals in farmland soils in this study area. As an artificial measure that strongly disturbed the soil environment, farmland consolidation was an important factor affecting the spatial distribution of soil heavy metal content. There were large differences in the content of heavy metals between farmland with different farmland consolidation measures and farmland in non-agricultural land consolidation areas, and the highest average values of various heavy metal content were in non-agricultural land consolidation areas. The content of heavy metals in farmland where building ditches, merging plots, land levelling, applying organic fertilizers, and comprehensive improvement were implemented was lower than that of non-agricultural land consolidation areas.
(5) The impact of heavy metals on bacterial community structure varied greatly under different levels of heavy metal pollution. Cultivated lands with low pollution levels were all located in farmland consolidation areas. A total of 4 bacterial phyla exhibited strong absorption and transfer functions for heavy metals such as Hg and Ni, and 6 bacterial genera showed a significant positive correlation with heavy metals such as Pb, As, and Ni. Most of the soil samples at the lightly polluted level were located in farmland consolidation areas. Among them, the bacteria Gemmatimonadetes and SBR1093 had strong adsorption and degradation functions on the heavy metals Cu and Pb. The bacteria genera *Gaiella, H16, Thiobacillus* and the heavy metals Pb, Cr, Ni content were significantly positively correlated. Among the soil samples with moderate pollution levels, 13 samples were located in farmland consolidation areas. A total of 11 bacterial phyla were significantly correlated with the heavy metals Pb, Cr, As, and Cd, respectively. Bacterial genera such as *Sphingomonas, Thioalkalispira*, and *Geothermobacter* were significantly positively correlated with the heavy metals Cr and Ni respectively. Among the soil samples with heavily polluted levels, a total of 7 samples were located in non-agricultural land consolidation areas. Among them, the heavy metals Cu, As, Ni, and Zn had significant effects on the 7 bacterial phyla. The bacterial genera *Geothermobacter, Bryobacter, Gemmatimonas, Nitrospira*, and *Bacillus* were significantly positively correlated with Cd, Pb, As, and Ni under the condition of consuming a lot of soil nutrients.

With the continuous increase of heavy metal pollution, the number of heavy metal-tolerant bacteria in the soil generally increased first and then decreased. This was probably because with the increase of pollution level, the vitality of heavy metal-tolerant bacteria was stimulated and better heavy metal adsorption and detoxification functions appear. However, as pollution continues to intensify, some strains were eliminated, leaving dominant strains. These strains, which had strong heavy metal absorption and detoxification functions in heavy metal polluted environments, could be used as effective bioremediation methods to improve the soil environment. They should be added to the cultivated land quality evaluation system to serve the cultivated land quality improvement project in farmland consolidation.

## Figures and Tables

**Table 1:** Table of soil bacterial diversity indicators under different farmland consolidation.

Land Consolidation Measures	The Community Richness Index		The Community Evenness Index		The Community Diversity Index		The Community Coverage
	Sobs	Chao	Shannoneven	Simpsoneven	Shannon	Invsimpson	Coverage
Comprehensive improvement	4312.00 ± 452.33a	5866.69 ± 523.61a	0.89 ± 0.01a	0.17 ± 0.02a	7.44 ± 0.12a	730.73 ± 118.61a	0.95 ± 0.01a
Applying organic fertilizers	4318.92 ± 447.21a	5768.07 ± 530.29a	0.88 ± 0.01a	0.15 ± 0.04a	7.36 ± 0.16a	658.19 ± 189.31a	0.96 ± 0.01a
Building ditches	4370.70 ± 443.19a	5808.02 ± 493.61a	0.88 ± 0.01a	0.16 ± 0.03a	7.39 ± 0.14a	693.77 ± 161.98a	0.95 ± 0.01a
Merging plots	4349.68 ± 440.11a	5823.19 ± 528.11a	0.88 ± 0.01a	0.15 ± 0.04a	7.38 ± 0.15a	675.83 ± 176.20a	0.96 ± 0.01a
Land levelling	4405.47 ± 396.52a	5963.16 ± 483.81a	0.88 ± 0.01a	0.16 ± 0.04a	7.39 ± 0.17a	696.92 ± 202.67a	0.96 ± 0.01a
Non-agricultural land consolidation	3338.20 ± 675.30b	4348.85 ± 922.49b	0.87 ± 0.01a	0.13 ± 0.04a	7.01 ± 0.25b	439.17 ± 166.72b	0.96 ± 0.01a

Note: The average value in the table is presented in the form of mean ± standard deviation. Different lowercase letters in the same column represent significant differences at the *p* < 0.05 level.

**Table 2:** Soil chemical properties among different groups in the study area.

Group	pH	Organic Matter (g/kg)	Available Phosphorus (mg/kg)	Available Potassium (μg/mL)	Total Nitrogen (g/kg)
Building ditches	7.09 ± 0.63b	46.63 ± 14.22b	90.78 ± 83.78a	29.65 ± 13.31a	2.41 ± 0.73b
Land levelling	7.23 ± 0.63a	41.97 ± 15.33b	72.85 ± 54.01b	28.99 ± 12.00a	2.20 ± 0.77b
Merging plots	7.11 ± 0.57b	41.52 ± 15.30b	70.24 ± 57.77b	28.60 ± 15.25a	2.23 ± 0.77b
Applying organic fertilizers	7.11 ± 0.64b	52.49 ± 11.59a	93.67 ± 84.50a	31.38 ± 14.57a	2.51 ± 0.64b
Comprehensive improvement	6.93 ± 0.51b	57.98 ± 11.66a	79.80 ± 58.37b	37.78 ± 7.61a	3.08 ± 0.45a
Non-agricultural land consolidation	7.38 ± 0.71a	30.21 ± 9.80c	53.43 ± 37.13c	24.00 ± 14.93b	1.69 ± 0.45c

Note: The average value in the table is presented in the form of mean ± standard deviation. Different lowercase letters in the same column represent significant differences at the *p* < 0.05 level.

**Table 3:** Three soil enzyme activities in the study area.

Group	Catalase (mg/g)	Phosphatase (mg/g)	Urease (mg/g)
Building ditches	207.66 ± 38.30a	16.017 ± 8.44a	0.25 ± 0.16a
Land levelling	203.62 ± 38.48a	17.35 ± 10.65a	0.28 ± 0.22a
Merging plots	201.61 ± 32.37a	15.28 ± 9.97b	0.23 ± 0.15a
Applying organic fertilizers	198.61 ± 41.72a	17.54 ± 9.68a	0.25 ± 0.16a
Comprehensive improvement	206.72 ± 28.98a	20.88 ± 10.58a	0.33 ± 0.16a
Non-agricultural land consolidation	183.58 ± 50.72a	12.32 ± 11.87c	0.22 ± 0.30a

Note: The average value in the table is presented in the form of mean ± standard deviation. Different lowercase letters in the same column represent significant differences at the *p* < 0.05 level.

**Table 4:** Soil heavy metal content in the study area.

Unit: mg/kg								
	Cu	Cd	Pb	Cr	As	Hg	Ni	Zn
Building ditches	45.95 ± 12.74	2.28 ± 0.23	43.39 ± 13.23	214.65 ± 11.67	4.66 ± 3.83	0.57 ± 0.28	53.15 ± 5.60	125.75 ± 26.70
Land levelling	39.44 ± 7.92	2.23 ± 0.27	37.54 ± 12.81	214.61 ± 11.34	4.21 ± 3.51	0.45 ± 0.15	54.06 ± 7.63	110.47 ± 18.39
Merging plots	41.72 ± 8.83	2.27 ± 0.33	37.62 ± 12.06	212.64 ± 11.22	4.99 ± 3.91	0.53 ± 0.25	54.55 ± 7.44	112.51 ± 21.58
Applying organic fertilizers	46.55 ± 12.25	2.27 ± 0.31	42.98 ± 10.38	216.45 ± 11.12	3.89 ± 2.90	0.53 ± 0.21	55.00 ± 6.86	128.36 ± 24.72
Comprehensive improvement	39.90 ± 9.17	2.28 ± 0.13	40.29 ± 14.98	208.70 ± 6.59	2.93 ± 3.94	0.47 ± 0.14	51.10 ± 6.93	117.82 ± 20.45
Non-agricultural land consolidation	48.97 ± 6.71	3.30 ± 0.55	46.83 ± 8.81	252.96 ± 11.05	7.88 ± 5.29	0.65 ± 0.37	64 ± 10.85	136.82 ± 21.17
Maximum	84	3.98	77.5	268.8	14.4	1.38	80.4	198
Minimum	25	1.57	20	195	0.81	0.1	39	70.6
Average value	45.17	2.5	41.94	223.79	5.29	0.55	56.77	123.27
Variation coefficient (%)	22.38	22.86	26.4	8.72	77.91	49.99	15.96	20.7
Background value	22.6	0.17	35.7	56	6.9	0.17	23.9	83.1

Note: The content of heavy metals in the table is presented in the form of average ± standard deviation.

## Supplementary Materials

The following supporting information can be downloaded at: https://www.mdpi.com/article/10.3390/ijerph19020845/s1, Supplementary S1: Layout of sample points; Supplementary S2: Soil index data.
